# Supplementary material for: Distinct neocortical mechanisms underlie human SI responses to median nerve and laser-evoked peripheral activation
Source: Imaging Neurosci (Camb). 2024 Feb 22;2:imag-2-00095. doi: 10.1162/imag_a_00095 (PMC12235563; doi:10.1162/imag_a_00095)
Supplement: Supplementary Material [file imag_a_00095-supp.pdf]

source → *target*	L2/3 Pyramidal	L5 Pyramidal	L2/3 Basket	L5 Basket
**L2/3 Pyramidal**	AMPA: 5e-4*<br>NMDA: 5e-4*	AMPA: 2.5e-4*<br>NMDA: n/a	AMPA: 5e-4<br>NMDA: n/a	AMPA: 2.5e-4<br>NMDA: n/a
**L5 Pyramidal**	n/a	AMPA: 5e-4*<br>NMDA: 5e-4	n/a	AMPA: 5e-4<br>NMDA: n/a
**L2/3 Basket**	GABA$_A$: 5e-2<br>GABA$_B$: 5e-2	GABA$_A$: 1e-3*<br>GABA$_B$: n/a	GABA$_A$: 2e-2<br>GABA$_B$: n/a	n/a
**L5 Basket**	n/a	GABA$_A$: 2.5e-2*<br>GABA$_B$: 2.5e-2*	n/a	GABA$_A$: 2e-2<br>GABA$_B$: n/a

Supplementary Table 1. Original local network parameters of the computational model prior to tuning. Each value represents a synaptic weight (µS) for an excitatory (i.e., AMPA or NMDA) or inhibitory (i.e., GABA$_A$ or GABA$_B$) connection. Asterisks denote parameters that were tuned from the default values shown here.

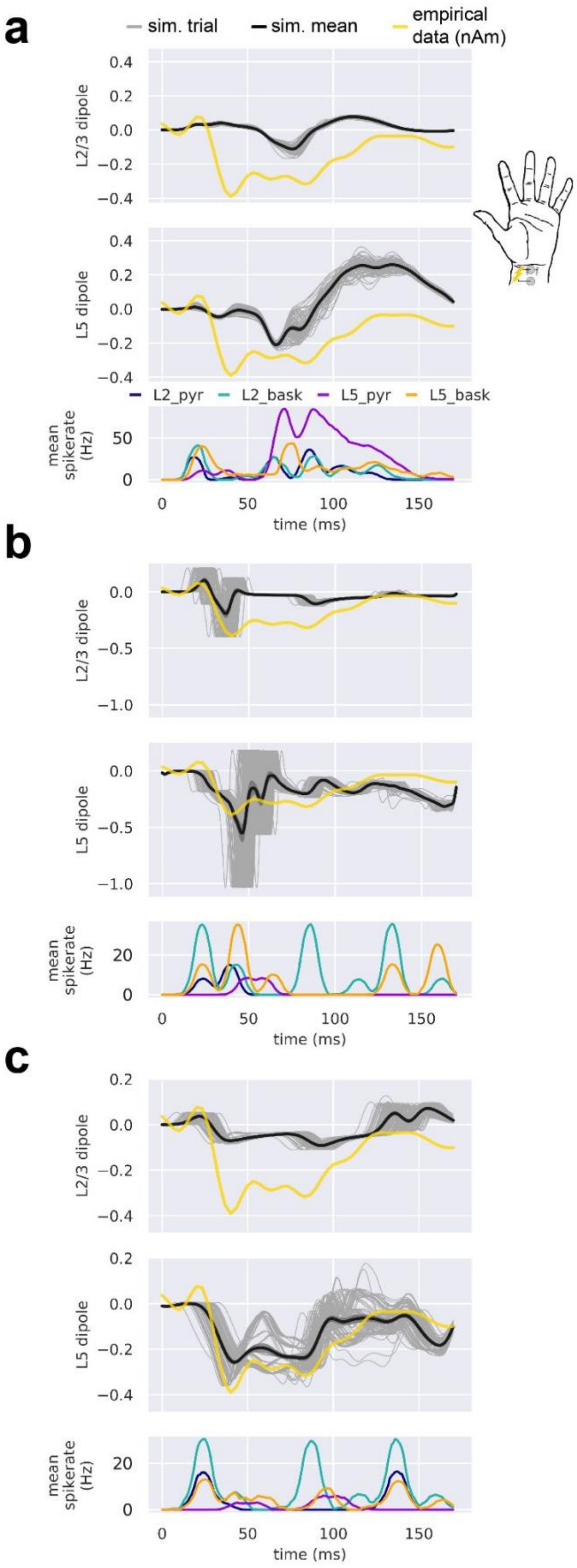

Supplementary Figure 1. Pyramidal neurons from L2/3 and L5 contribute differently to the simulated current dipole of the MN response. From top to bottom, each panel contains the simulated dipole (individual

trials, grey traces n=100; trial-mean, black trace) from L2/3 pyramidal neurons alongside the empirical MN response (yellow trace), the simulated dipole from L5 pyramidal neurons, and the population-mean spikerate (i.e., the average spikerate of a neuron of a given type). Panels (a-c) correspond, respectively, to Figure 4a-c.

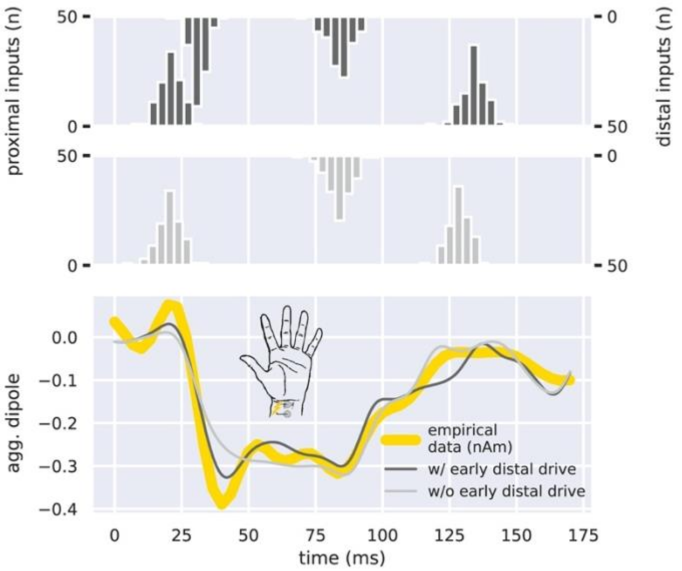

Supplementary Figure 2. Removal of the early distal drive at ~30 ms removes the model's ability to produce a distinct MN P1 deflection. Using numerical optimization to tune all parameters of the drive sequence either including an early distal drive or excluding an early distal drive, only the former is able to produce a distinct P1 deflection in the simulated average current dipole (n=100 trials, each smoothed with a 20 ms Hamming window and scaled by a factor of 40).

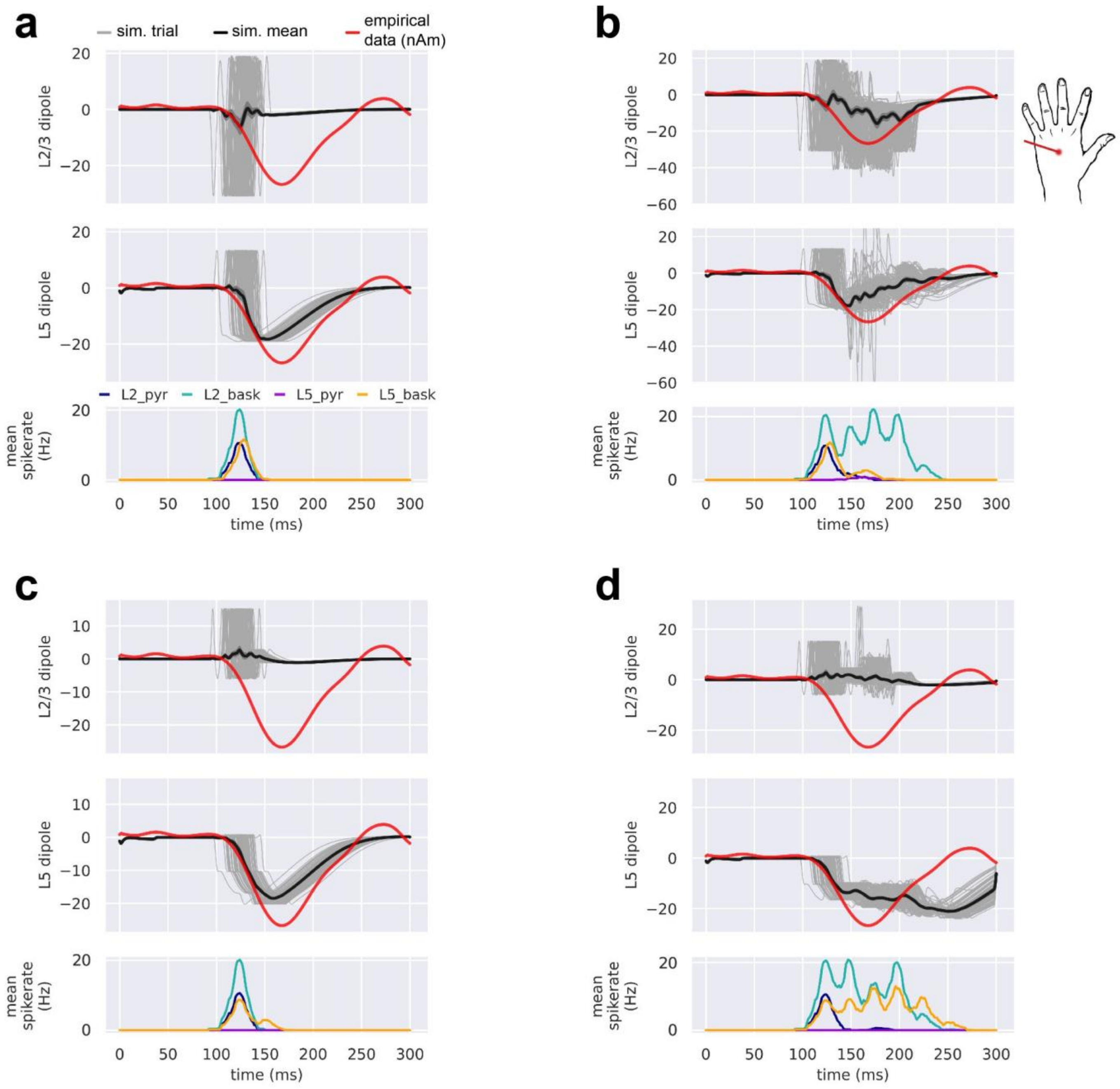

Supplementary Figure 3. Pyramidal neurons from L2/3 and L5 contribute differently to the manually-tuned simulated current dipole of the LE response. From top to bottom, each panel contains the simulated dipole (individual trials, grey traces n=100; trial-mean, black trace) from L2/3 pyramidal neurons alongside the empirical LE response (red trace), the simulated dipole from L5 pyramidal neurons, and the population-mean spikerate (i.e., the average spikerate of a neuron of a given type). Panels (a-c) correspond, respectively, to Figure 7a-c.

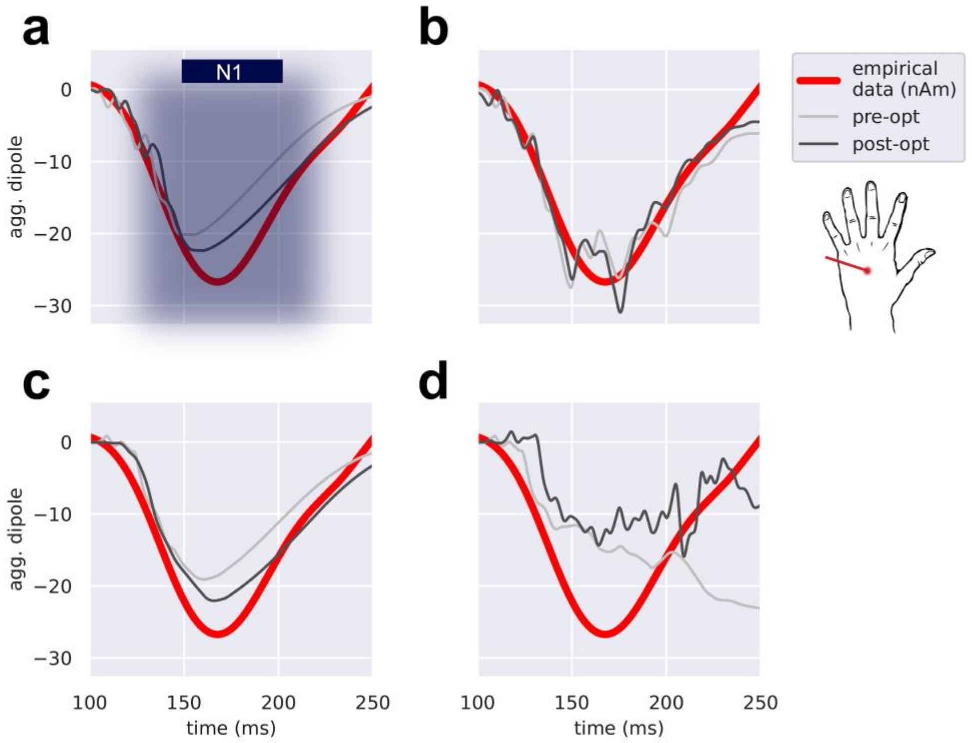

Supplementary Figure 4. Parameter optimization provides minimal improvement to the manually-tuned drive configurations shown in Figure 7. Except for the case with repetitive distal drives (b), the trial-average aggregate current dipole before (pre-opt, grey) and after (post-opt, black) optimization for all other drive configurations including a single distal drive (a), a single proximal drive (c), and repetitive proximal drives (d) fail to simulate the depth and breadth of the empirical LE N1 deflection (red).

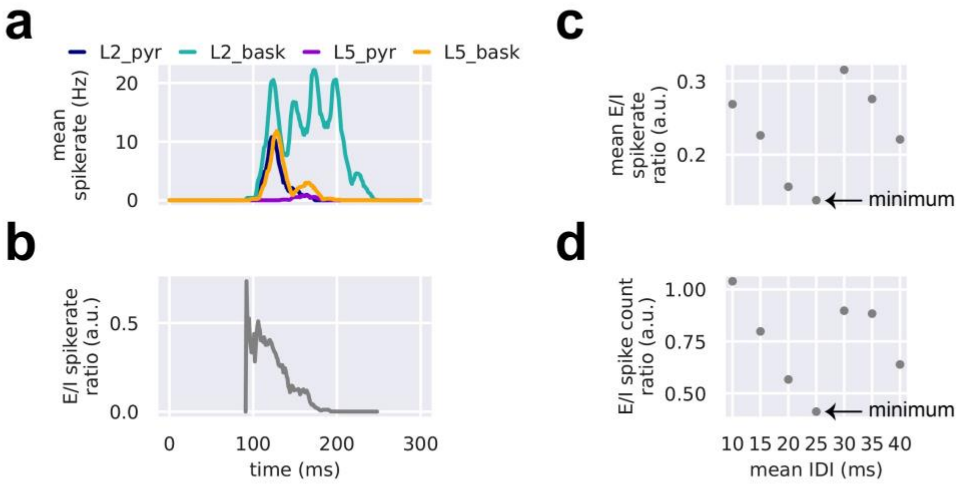

Supplementary Figure 5. A burst of repetitive distal drives delivered at mean intervals of 25 ms minimizes network excitation relative to network inhibition. Such an excitation/inhibition (E/I) balance maximizes downward current flow in the pyramidal apical dendrites while preventing runaway excitation. (a) With a mean 25 ms inter-drive-interval (IDI), repetitive distal drives recruit large amounts of L2/3 inhibitory basket cell spiking at corresponding intervals while largely diminishing the spikerate of other (i.e., L2/3 pyramidal, L5 pyramidal, and L5 basket) cell populations. (b) Also with a mean 25 ms IDI, the ratio of the aggregate mean excitatory (L2/3 + L5 pyramidal) cell spikerate divided by inhibitory (L2/3 + L5 basket) cell spikerate is always less than 1 and attenuates with successive distal drives from 120-195 ms. (c) Across simulations with mean IDIs ranging from 10-40 ms, the time-average E/I spikerate ratio is minimal with an IDI of 25 ms. (d) Same as (c), except instead of mean E/I spikerate ratio, we show the total E/I spike count ratio across all cells, all trials, and all time of the simulation.

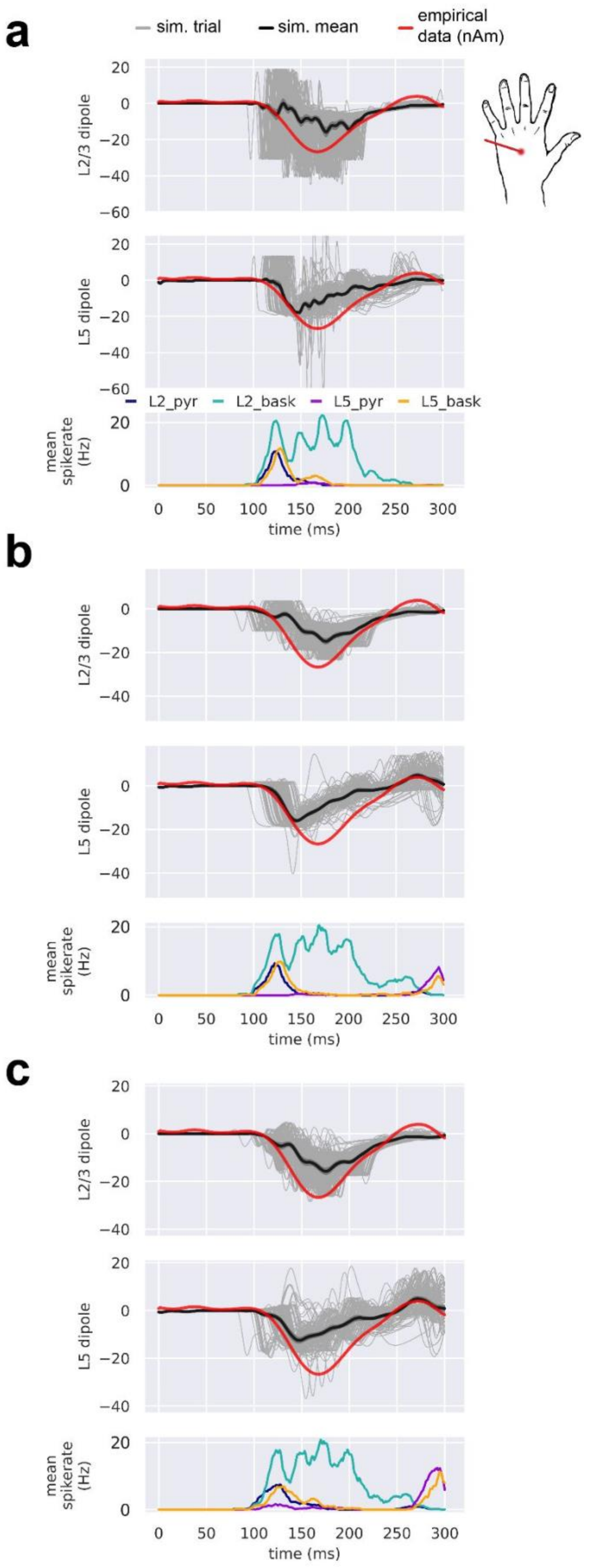

Supplementary Figure 6. Pyramidal neurons from L2/3 and L5 contribute differently to the optimized simulated current dipole of the LE response. From top to bottom, each panel contains the simulated dipole

(individual trials, grey traces n=100; trial-mean, black trace) from L2/3 pyramidal neurons alongside the empirical LE response (red trace), the simulated dipole from L5 pyramidal neurons, and the population-mean spikerate (i.e., the average spikerate of a neuron of a given type). Panels (a-c) correspond, respectively, to Figure 9a-c.

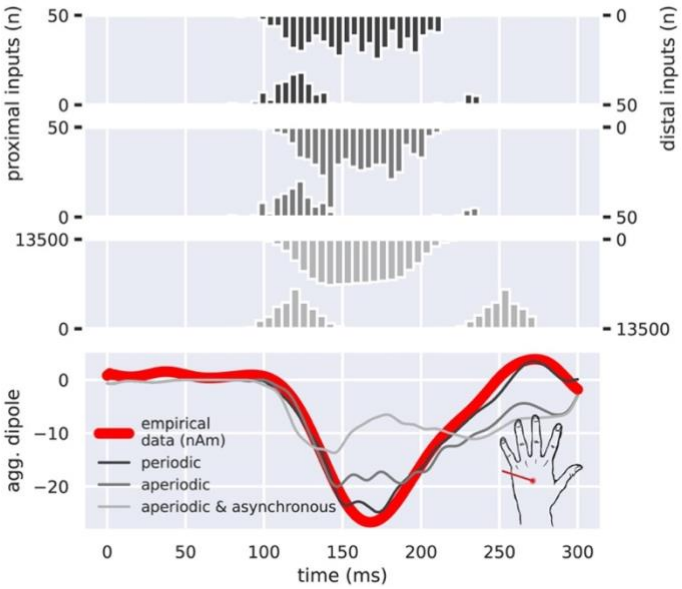

Supplementary Figure 7. Periodicity within the burst of distal drives is essential for reproducing the LE N1 deflection. The final optimized drive configuration shown in Figure 9c includes a burst of 40 Hz periodic distal drives (top histogram). Removal of periodicity within the burst of drives, for either synchronous (middle histogram) or asynchronous (bottom histogram) drives by uniformly sampling distal drive times within the burst window (120-200 ms) only impairs the model's ability to generate a LE N1 deflection that is both deep and has a subsequent rebound at ~250 ms (bottom current dipole response plots). Consistent with other simulations of this study, each simulated current dipole shown here is the aggregate average current dipole of n=100 trials, each smoothed with a 20 ms Hamming window and scaled by a factor of 2500.
